# Secondary psychosis after a temporal lobe resection in a patient with refractory epilepsy. A case report

**DOI:** 10.1192/j.eurpsy.2021.2066

**Published:** 2021-08-13

**Authors:** A. Cerame Del Campo, A. Franco Soler, P. Coucheiro Limeres

**Affiliations:** Centro De Salud Mental, Instituto Psiquiátrico José Germain, Leganes, Spain

**Keywords:** temporal lobe resection, secondary psychosis, temporal lobe epilepsy

## Abstract

**Introduction:**

We present the case of a 34-year-old female patient with no prior psychiatric record who was treated in our outpatient department due to persecutory delusions of recent onset. The patient had a history of refractory temporal epilepsy since adolescence and underwent a temporal lobe resection 4 month prior to the appearance of her symptoms.

**Objectives:**

Temporal lobe resection is a well-established technique to treat refractory temporal lobe epilepsy in which psychotic symptoms are an infrequent complication; the most frequent being cognitive sequelae, visual field defects and depression. According to several sources, this symptomatology may be underdiagnosed and undereportend and there have been a number of case reports and series of cases which describe the aforementioned entity.

**Methods:**

A case report is presented alongside a review of the relevant literature regarding cases of secondary psychosis after brain surgery.

**Results:**

During her treatment we administered olanzapine up to doses of 7.5mg per day because of the risk of reducing the convulsive threshold. We observed a marked improvement and the disappearance of the delusions. The dose of olanzapine has been maintained for a year with no important side-effects and without a relapse in symptoms.

**Conclusions:**

Psychotic symptoms as a complication of temporal lobe resection may be more frequent than what was thought in the past. It is important to study this phenomenon more in-depth because the symptoms may remain undetected and present worse outcomes given that there are effective treatments which could ameliorate the condition.

**Disclosure:**

No significant relationships.

